# A Reliable Hemostatic

**Published:** 1922-08

**Authors:** 


### A RELIABLE HEMOSTATIC

But few remedies have proved as reliably efficacious as has Thromboplastin Squibb in cases of bleeding the site of which can be reached by the product; and in dental practice it often produces results little short of miraculous.

Thromboplastin Squibb is a true physiologic hemostatic prepared from cattle brain in accordance with the directions of Dr. Alfred F. Hess, connected with the Research Laboratory of the New York City Department of Health. It contains the lipoid substances of brain tissue described by Hirschfelder, in physiological solution of sodium chloride and preserved with 0.3 per cent. of tricresol.

In his first clinical report Hess [1] stated that Thromboplastin had been found serviceable for local use in hemorrhage, especially in bleeding associated with true

[1] "Tissue Extract as a Hemostatic": *Jour. Amer. Med. Ass'n,* 1915, lxiv, p. 1396.

hemophilia. Some months later a paper was published by Dr. John J. Cronin,[2] on the routine application of Thromboplastin as evolved by Hess, in 2036 tonsil and adenoid operations performed in the clinics of the New York Department of Health. He found the product of decided value in decreasing bleeding and the incidence of undue hemorrhage. He says:

"Not in a single instance has the operator or nurse been compelled to return to the clinic to care for a bleeder after operation since the use of Thromboplastin was made a routine procedure. Thromboplastin is safe, effective and easily applied."

In a second paper Hess[3] embodies the clinical results obtained with Thromboplastin in maternity hospitals where it was employed locally in cases of melena neonatorum, in bleeding from the cord, vagina, skin, mouth, etc., after circumcision, etc. The results were more than satisfactory as well as lasting, even where other measures previously tried were of no avail. The conclusions drawn are as follows:

"Thromboplastin solution has proved itself of practical value in controlling hemorrhage wherever it can reach the site of bleeding. In cases of true hemophilia it may be regarded almost as a specific hemostatic. It is to be recommended for local use in the bleeding of the new-born, in nasal hemorrhage, and in the parenchymatous bleeding associated with various operations, etc. Where local applications fail, it should be injected into the site of hemorrhage, as in bleeding from the gums following tooth extraction. * * * Further clinical experience is necessary before its value can be determined

---

[2] "Thromboplastin as a Hemostatic": *Jour. Amer. Med. Ass'n*, 1916, lxvi, p. 557.

[3] "A Further Report on Thromboplastin Solution as a Hemostatic": *Jour. Amer. Med. Ass'n*, 1916, lxvii, p. 1717.

as a hemostatic in connection with hemorrhage from the gastro-intestinal tract. However, it is innocuous when given by mouth in considerable dosage, and would seem to be indicated in bleeding from the stomach and from the upper intestine. In addition to its hemostatic action this tissue extract (Thromboplastin) has been found to possess healing properties, actively stimulating granulation tissue and hastening epithelization. It is therefore applicable as a dressing for torpid ulcers and for sluggish wounds."

It may be of advantage to note a few other conditions in which Dr. Hess' records show that Thromboplastin has been successfully used. In this connection may be mentioned the nasal hemorrhage associated with toxic conditions, more especially diphtheria, for which it has been employed extensively at the Willard Parker Hospital for Contagious Diseases in New York; and likewise for nasal hemorrhage complicating the primary blood diseases, for example, in acute leukemia; furthermore, at operations for hepatic bleeding, in hemorrhage from the bladder, in operations involving the spinal cord, in prostatic operations, and in bronchoscopy for the removal of diphtheritic membrane or old foreign bodies with bleeding granulation tissue.

As regards the use of Thromboplastin Squibb in obstinate bleeding following tooth extractions, Dr. Will J. Brownell, of Chicago, Ill., writes as follows:[4]

"In the selection of a hemostatic, one that is physiological in its action, does not constrict tissue with a subsequent relaxation and secondary hemorrhage, and can be relied upon to act quickly and with no systematic reaction, is the ideal agent. After much investigation, which was for personal reasons as much as any, for I am a 'bleeder,' I stumbled upon a hemostatic that filled

[4] Letter to the Medical Department of E. R. Squibb & Sons.

all requirements, and that at a time when I most needed it, for I had been bleeding at intervals for ten days following the extraction of a molar. I first applied the product, *i. e.*, Thromboplastin Squibb, locally; but, owing to the systemic condition, failed to get any results. A representative of the Squibb Research Laboratory dropped into the office about this time and suggested that I follow out a regular routine recommended in such cases as mine. This I did; a second molar was extracted on left side (upper), and regular routine followed with extra precautions in the way of firm packing with astringents, my condition being known. The packing was held firmly in place by a wire from the third molar to the first molar. As I expected, an oozing continued off and on for twenty-four hours, when the first packing was removed and a fresh one put in, also Monsel's solution applied; various other styptics were applied, but to no avail.

"On the tenth day Thromboplastin Squibb was suggested, and I applied it locally, with a cessation of bleeding temporarily. However, about 4 p. m. the oozing began afresh.

"About this time the man from the Research Laboratory came again, and from him I obtained the routine for the use of Thromboplastin in stubborn cases of bleeding. Following this closely, I had the gum tissue injected with 3 Cc. of the *hypodermic* type of Thromboplastin, which was effective until about 11:30 p. m., when a slight oozing was noticed. I then had 20 Cc. injected *subcutaneously* in the abdominal region, and at 12:30 another 20 Cc., although the physician in charge says that the second was not necessary. The bleeding ceased and there has been no further recurrence. The socket is healed and is healthy looking.

"Although decidedly skeptical, I must admit that I was more than agreeably surprised. Since my own case I have used it on a 'bleeder' in the office, with brilliant results. I am submitting this report and would like to hear from others of the profession regarding Thromboplastin Squibb."

Another professional gentleman writes as follows:[5]

"Most hemostatic agents act as *blood coagulants*, forming insoluble precipitates with the proteins of the blood and are transient in effect. Epinephrine (adrenalin) preparations act upon the capillary walls as temporary constricting agents whose effect lasts only for a short time, and is followed by a dilatation of the blood vessels, and, frequently, secondary hemorrhage hours after the patient is beyond the care of the dentist.

"When blood fails to clot quickly, its clotting time can be hastened by the addition to the blood of physiological substances which contribute to the normal formation of a clot. Concentrated extracts of tissue juices when added to fresh blood shorten its clotting time very markedly. Extract of brain tissue causes this effect most strikingly. Thromboplastin (Squibb) is an extract of brain tissue (ox brain), which acts physiologically and not chemically. It maintains its power to hasten clotting for a considerable time. In local hemorrhage it acts on direct application to the bleeding surface, applied on cotton or gauze. Upon the blood of chronic bleeders (hemophiliacs) it is *specific* in its effect.

"Following the extraction of teeth, Thromboplastin has shown its value in preventing prolonged bleeding, by packing with cotton saturated with a few drops of Thromboplastin local."

Thromboplastin Squibb is applied pure direct to the bleeding surface by means of cotton, gauze or a tampon.

---

[5] Personal communication to the EDITOR.

Emphasis is to be laid on the necessity of having it come into direct contact with the bleeding point; if soft clots have formed, they should be removed. Furthermore, although a cursory application in many instances will suffice to hasten coagulation, where such does not occur, the hemostatic should be held in close contact with the bleeding surface for some minutes. From time to time cases have been encountered in which attention to this detail has checked hemorrhage which had seemed uncontrollable.

Where injection hypodermically or into the gums is indicated, as in internal parenchymatous hemorrhage, in hemophilia, and obstinate bleeding following tooth extraction, Thromboplastin *Hypodermic* Squibb should be used; for all topical uses, the regular (local) Thromboplastin would be employed. Both forms are marketed in 20 Cc. vials, which should be kept in a *cool* place, preferably in the refrigerator.

The dose of Thromboplastin Squibb in gastric hemorrhage is 20 Cc., taken with a glass of water several times daily. In parenchymatous bleeding and in hemophiliacs the hypodermic variety is administered subcutaneously or intravenously in quantities of 10 Cc. to 20 Cc. every twenty-four to seventy-two hours.

